# Membrane Distillation: Pre-Treatment Effects on Fouling Dynamics

**DOI:** 10.3390/membranes11120958

**Published:** 2021-12-03

**Authors:** Paula G. Santos, Cíntia M. Scherer, Adriano G. Fisch, Marco Antônio S. Rodrigues

**Affiliations:** 1 Graduation Program in Environmental Quality, Universidade Feevale, Novo Hamburgo 93525-075, Brazil; paula.goncalves@feevale.br; 2 Chemical Engineering Department, Universidade Feevale, Novo Hamburgo 93525-075, Brazil; cintiascherer@feevale.br; 3 Chemical and Materials Engineering Department, University of Alberta, Edmonton, AB T6G 2R3, Canada; 4 Graduation Program in Technology of Materials and Industrial Processes, Universidade Feevale, Novo Hamburgo 93525-075, Brazil; marcor@feevale.br

**Keywords:** membrane distillation, fouling, petrochemical effluent, pre-treatment, water recovery

## Abstract

In the research reported in this paper, membrane distillation was employed to recover water from a concentrated saline petrochemical effluent. According to the results, the use of membrane distillation is technically feasible when pre-treatments are employed to mitigate fouling. A mathematical model was used to evaluate the fouling mechanism, showing that the deposition of particulate and precipitated material occurred in all tests; however, the fouling dynamic depends on the pre-treatment employed (filtration, or filtration associated with a pH adjustment). The deposit layer formed by particles is not cohesive, allowing its entrainment to the bulk flow. The precipitate fouling showed a minimal tendency to entrainment. Also, precipitate fouling served as a coupling agent among adjacent particles, increasing the fouling layer cohesion.

## 1. Introduction

Access to water has sustained the development of human society. Irrigation was as essential for the development of ancient civilizations as it is today for modern society, considering the demand for food which is imposed by a constantly growing world population. In addition to food, modern human society also demands the diversified manufacturing of goods to maintain its lifestyle. Regardless of the final product, industrial processes usually employ a large amount of water to convert the raw materials into proper goods for human consumption. This features the petrochemical and chemical companies, which have predominantly used water from natural resources, reducing its availability for nobler uses, such as the production of potable water. Indeed, the generation of pollutants by the manufacturing processes and their disposal into nature have also negatively impacted the quality of the water [1,2]. Due to the growing demand, water becomes a scarce natural resource for both domestic and industrial users, mainly for countries with developing economies where larger demands of water are needed to sustain industrialization [3,4].

Companies that are aware of the water scarcity problem have invested in projects to enhance the production process towards sustainability. The scope of such projects typically encompasses either modification of the manufacturing process to reduce the amount of water used in [5,6], the recovery of water from the industrial effluent or tailings and its reuse in the production process [2,7], or the use of water from alternative sources [8]. In this sense, the use of membrane separation technologies for the treatment of industrial wastewater, which aim to recover and to reuse water, has been the subject of multiple academic and industrial studies [9,10,11,12,13]. Due to a large demand for water by petrochemical companies, water recycling has been studied using hybrid membrane processes such as electrodialysis reversal-membrane distillation (EDR-MD) [14,15,16] and osmosis reversal-membrane distillation (OR-MD) [14,17]. In these examples, membrane distillation has been employed to recover water from a highly saline effluent generated by the precedent membrane separation process. Consequently, the association of membrane distillation has augmented the amount of water that is recovered from industrial wastewater, resulting in a reduction of the freshwater demand from natural resources. However, fouling has negatively affected membrane distillation performances when processing highly saline effluents, by limiting both the recovery rate of water and its quality. Although some strategies for the mitigation of fouling have been studied, such as pre-treatment of the saline effluent [18], through the development of membranes and modules [19,20], and the optimization of operational conditions [21], fouling is still a concern in membrane distillation and there is no unique solution for this issue. As fouling phenomena are intrinsically dependent on the nature of the effluent [7,22,23,24,25,26,27,28] and are time-dependent, dynamic experimental studies are needed to shed light on fouling mechanisms and their features.

Primarily petrochemical companies produce saline effluents, which tend to generate inorganic fouling when processed by membrane distillation. Usually, for this kind of effluent, the deposit layer is formed by precipitate and particulate materials. The mechanism of adherence of these foulants on the membrane surface is driven by the balance of van der Waals and electrical double layer forces [7,22,26,27,28,29]. Although studies have disclosed information regarding the mechanisms of fouling formation for distinct effluents, the dynamics of material accumulation on the membrane surface are still not clear for membrane distillation. For example, the impact of the entrainment, layer cohesivity, and the nature of the membrane surface (pristine or washed membrane) on the layer build-up over time remain as research questions in the development of membrane distillation technology. Indeed, studies of fouling dynamics are fundamental for the design and sizing of membrane distillation units, as they impact the determination of the filtration area, for example.

Aiming to contribute to the increases in knowledge about fouling for the design, sizing, and operation of industrial-scale membrane distillation equipment, the present work reports about the dynamic of fouling deposition occurring during the membrane distillation processing a concentrated saline effluent. Specifically, there is no research on fouling formation by dynamic mathematical modeling, to the best of the authors’ knowledge. In this paper, a set of equations for the rates for deposition and entrainment is proposed, based on fouling mechanisms disclosed in the literature [26,27,28,29]. The experimental data used in the present research were obtained from a bench-scale membrane distillation and were analyzed using a phenomenological mathematical model. The influence of pre-treatments of the saline effluent on the dynamic of fouling is also reported.

## 2. Materials and Methods

**Figure 1:**
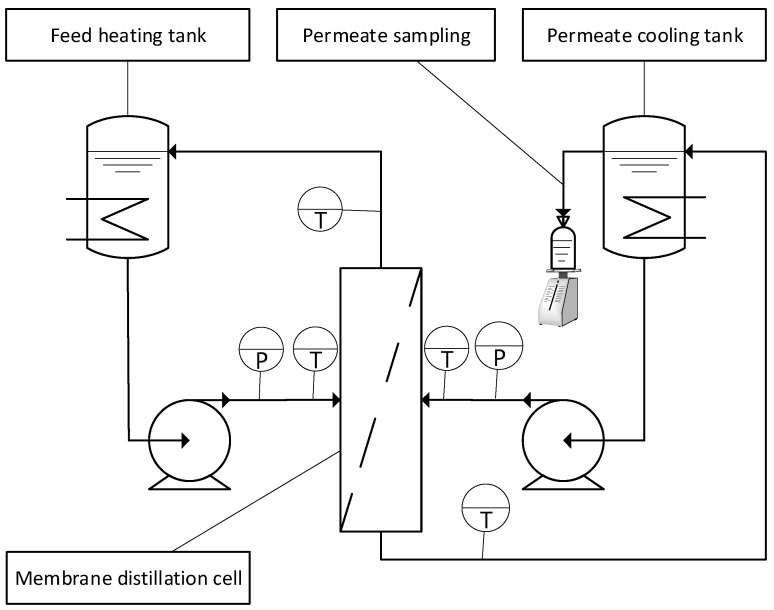
Experimental setup of the bench-scale direct contact membrane distillation.

The experimentation was conducted in a bench-scale direct contact membrane distillation (Figure 1). The permeate and retentate streams were set in the concurrent direction, and the flow was parallel to the membrane surface. Details about the membrane used in the experiments are given in Table 1.

The membrane distillation tests were conducted using a high saline effluent, obtained as the concentrated discharge of an electrodialysis reversal (EDR) plant, which has been used to treat petrochemical wastewater. This effluent is composed of high concentrations of suspended solids, calcium, iron, magnesium, silica, sodium, chloride, and sulfate, as presented in Santos et al. [7]. Tests were conducted using this concentrated saline effluent as received, after filtration, and after filtration and pH adjustment (pH 4.5–5.0), which occurred during the processing time as well. Filtration was performed using a paper filter with a pore size ranging from 4 to12 µm. The solution pH was adjusted using concentrated HCl.

Each membrane distillation test lasted about 156 h (9400 min) and was divided into 3 parts: membrane distillation with a new membrane (pristine) (72 h), membrane cleaning (12 h), and membrane distillation with a cleaned membrane (72 h). For the sake of simplicity, the membrane cleaning was performed using flowing, hot (about 57 °C), demineralized water throughout the system for 12 h. No previous studies were conducted to optimize these cleaning conditions.

During a typical experiment, the transmembrane flux was measured every 30 min, which is reported as an average of 3 measurements. Concomitantly, temperature and pressure data were collected as well, and reported in this study as an average of 3 measurements. The experimental standard deviation for transmembrane permeate flux was ν_exp_ = 0.04 kg m^−2^ s^−1^. Each experiment was reproduced 3 times and are reported as an average with a standard deviation less than that of the transmembrane permeate flux measurement. Typical operational conditions of the membrane distillation experiments are listed in Table 2.

A complete physical–chemical characterization of the effluent, as well as the permeate obtained by membrane distillation and the microanalysis (SEM-EDS) of fouled membrane surfaces, are found in the work of Santos et al. [7].

## 3. Mathematical Modeling

**Figure 2:**
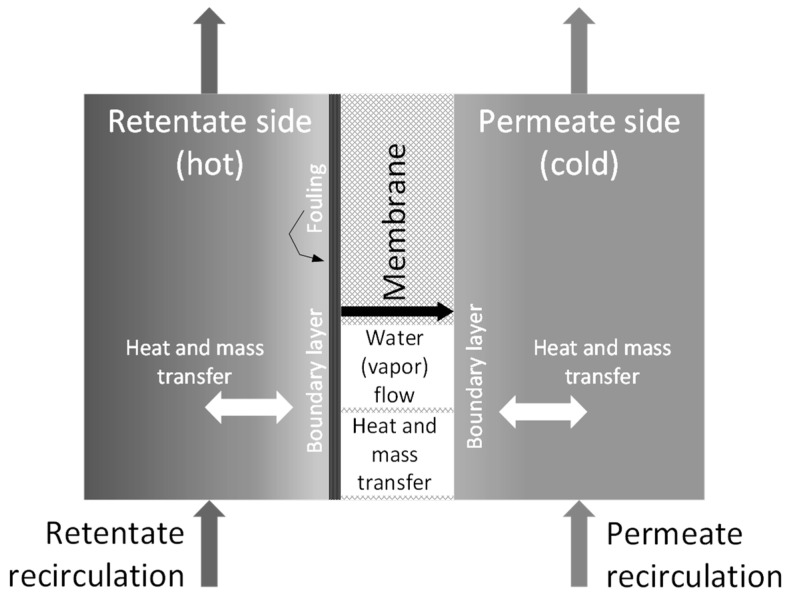
Modeling scheme for the membrane distillation cell.

A phenomenological lumped mathematical model of the bench-scale membrane distillation cell was built by coupling mass and heat balances. For the sake of an easy reading of the article, the resulting system of equations and its implementation are detailed in the Supplementary Material. The assumptions that were taken into account for the model development were: (I) membrane distillation cell is a closed system, (II) convective transfer of heat and mass between bulk flow and membrane surface occurs in both retentate and permeate sides, (III) transport of water vapor in the membrane pores occurs through stagnant air, and (IV) conductive heat transfer occurs through both the membrane skeleton and water vapor within pores. The schema in Figure 2 illustrates the assumptions used to model the bench-scale membrane distillation cell.

### 3.1. Model Prediction Capability

The model predicts the transmembrane flux, the temperature and concentration on the membrane surface, and the temperature of the outlet streams of the cell (retentate and permeate). As the heating and cooling recirculation streams were not included in the model, the inlet temperature of both retentate and permeate streams needed to be informed to allow the model calculation. Fouling resistance was also a parameter.

### 3.2. Assessment of Fouling Resistance

**Figure 3:**
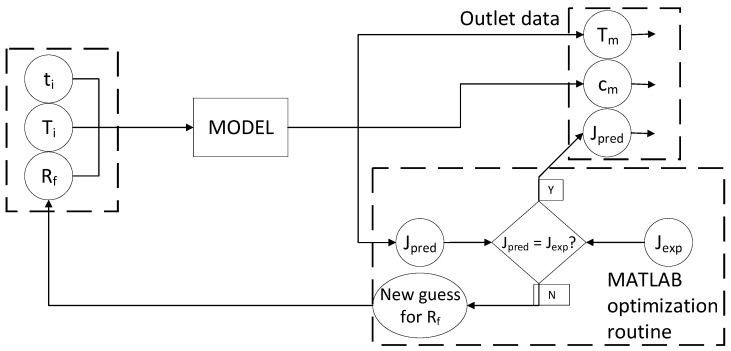
Optimization routine for the assessment of fouling resistance.

The assessment of the fouling resistance (*R_f_*) occurring in the membrane distillation was accomplished using an optimization routine (Figure 3), in which the quadratic error between the predicted and experimental data of transmembrane flux was minimized. Only for the first experimental point, when *R_f_*~0, were the mass transfer coefficients adjusted by minimization of the quadratic error of cell outlet temperature for the retentate and permeate recirculation streams concomitant to the transmembrane flux. 

The procedure in Figure 3 was performed for every experimental data collected during the experimentation, allowing us to obtain a time-dependent profile for fouling formation.

### 3.3. Dynamic of Fouling Deposition

A multitude of theoretical approaches is available in the literature to describe the formation of fouling in membrane separation processes [26,29,31,32,33,34,35]. Fundamentally, the applicability of a given approach relies on the nature of the material that is deposited or formed on the membrane surface, as well as on the interfacial properties between the potential foulant and the surface. In the present study, the typical composition of the saline effluent that was processed by membrane distillation was assessed to properly model the fouling formation [7]. According to the physical–chemical characterization of the effluent, fouling can potentially occur due to the deposition of suspended particles, as well as the precipitation of insoluble compounds on the membrane’s surface. Fouling due to surface reactions, corrosion, or biological material were not considered probable.

The deposition rate of suspended particles onto the membrane’s surface is driven by the concentration difference existing between the bulk flow and the membrane vicinity. A convective model can be used to describe the rate of deposition, as given in Equation (1),

(1) $$I_{part}=K_{part}\left(c-c_{m}\right)=K_{part}c$$

where: *I_part_* is the deposition rate of the suspended particles (m^−1^), *K_part_* is the effective mass transfer constant (m^2^ kg^−1^), *c* is the concentration of particles in the retentate bulk flow (kg m^−3^), and *c_m_* is the concentration of particles on the membrane vicinity (kg m^−3^). Commonly, *c_m_* is null, assuming that all particles in the membrane vicinity instantly adhere to the membrane surface.

The deposition rate of precipitates was assumed to be a function of the concentration of sites on the membrane’s surface from which the insoluble compound can nucleate and accumulate [27,29,36]. According to this theoretical approach, materials already adhered to the membrane’s surface are expected to play the role of nucleation sites. In this regard, Equation (2) is proposed to model the deposition rate of precipitates,

(2) $$I_{ppt}=K_{ppt}R_{f}{}^{n}$$

where: *I_ppt_* is the deposition rate of precipitates (m^−1^), *K_ppt_* is the effective constant, *R_f_* is the fouling resistance (s m^−1^), and *n* is the rate order. The unit of *K_ppt_* depends on the rate order (*n*).

Deposited material that is weakly adhered could eventually be removed from the deposited layer by shear forces [37]. The removal rate of adhered materials from the membrane surface (entrainment) could be assessed as given in Equation (3),

(3) $$I_{cis}=K_{cis}\tau R_{f}$$

where: *I_cis_* is the entrainment rate of materials (m^−1^), *K_cis_* is the effective constant (m s kg^−1^), *R_f_* is the fouling resistance (s m^−1^), and *τ* is the shear rate induced by the flow (kg m^−1^ s^−2^), which is assessed in Equations (4)–(6).

(4) $$\tau =f\rho_{w}v^{2}$$

(5) $$f=\frac{64}{N_{Re,f}},\;\mathrm{for}\;\mathrm{laminar}\;\mathrm{flow}$$

(6) $$v=\frac{G_{f}}{\rho_{w}A_{s}},\;\mathrm{for}\;\mathrm{laminar}\;\mathrm{flow}$$

where: *f* is the friction factor, *ρ_w_* is the specific mass of the retentate solution (kg m^−3^), *v* is the flow velocity (m s^−1^), *N_Re,f_* is the Reynolds number of the retentate flow in the cell, and *A_s_* is the transversal section area for the retentate flow (m^2^).

Then, the dynamic balance for the fouling resistance, *R_f_*, could be assessed by coupling the aforementioned rates of deposition and entrainment, as given in Equation (7),

(7) $$\frac{dR_{f}}{dt}=I_{part}+I_{ppt}-I_{cis}$$

The parameters in Equations (1)–(7), *K_part_*, *K_ppt_*, *n*, and *K_cis_*, were regressed using the fouling resistance profile obtained using the routine detailed in Figure 3.

## 4. Results and Discussion

The analysis of the fouling mechanism occurring in the membrane distillation process was performed using the experimental data and mathematical modeling. The use of the proposed model allowed us to understand the phenomena that cannot be experimentally measured.

### 4.1. Experimental Data and Fouling Resistance Assessment

**Figure 4:**
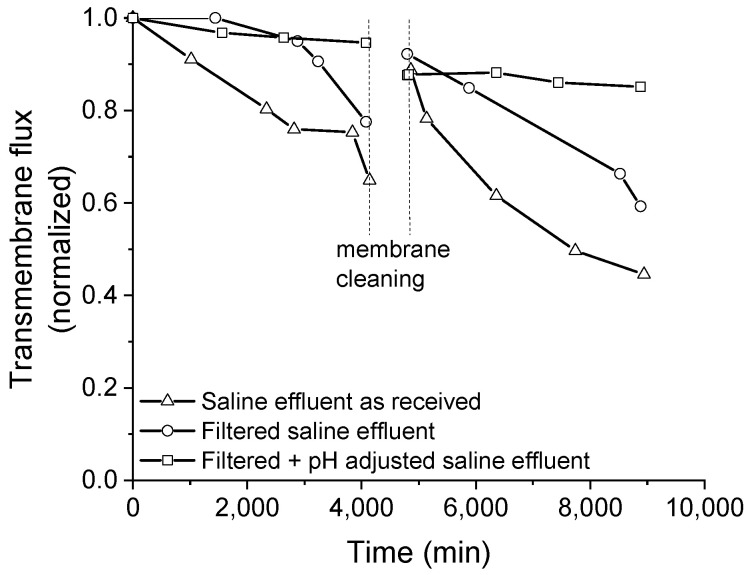
Experimental transmembrane fluxes.

The normalized transmembrane flux that was obtained for the membrane distillation tests using the saline effluent as received (i.e., without pre-treatment), filtered, and filtered with pH adjusted (pH ~ 5) are shown in Figure 4.

The transmembrane flux markedly dropped when the saline effluent was processed as received (line with triangle marks in Figure 4). The drop in the transmembrane flux was delayed when filtration was employed to treat the saline effluent before processing it by membrane distillation (line with circle marks in Figure 4). However, when filtration was associated with the pH control of the effluent over the processing time (line with square marks in Figure 4), the drop in transmembrane flux was seven times smaller when compared to the case without pretreatment, which remained somewhat steady up to 4320 min.

After cleaning the membrane by recirculating deionized water for 12 h (the period between vertical dash lines in Figure 4), a new sample of the saline effluent was processed for 72 h. In all tests, the initial value of the dimensionless transmembrane flux that was obtained after cleaning the membrane was similar (about 0.9 or 90%), as evidenced in Figure 4. This result suggests that some sort of fouling was not successfully removed by the cleaning procedure, reducing the membrane permeability for further uses by 10% in comparison with the pristine membrane.

After cleaning, the processing of the as-received effluent presented the highest drop in the transmembrane flux. When the effluent was filtered, the drop in the transmembrane flux was reduced, there was no evidence of any induction period occurring such as was seen with the pristine membrane. The lowest drop in the transmembrane flux after the membrane cleaning was obtained when the filtered effluent was processed with pH control. These results provide evidence that the physical treatment (filtration) and physicochemical treatment (filtration + pH adjustment) dispensed to the saline effluent positively influenced the maintenance of the transmembrane flux during the membrane distillation process before and after membrane cleaning.

**Figure 5:**
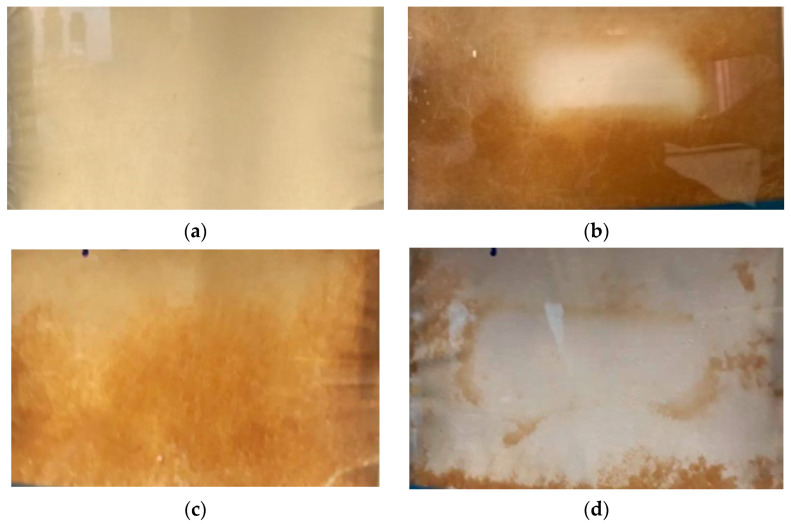
Fouling during membrane distillation. Pristine membrane (**a**), fouled membrane processing saline effluent as received (**b**), fouled membrane processing filtered saline effluent (**c**), and fouled membrane processing filtered, and pH adjusted saline effluent (**d**).

According to Figure 5, the pre-treatments reduced the formation of fouling on the membrane surface. A sparse deposition was observed on the membrane surface when associating filtration with pH adjustment, as a pre-treatment of the effluent, which allowed us to obtain a steady transmembrane flux over time.

It is worth mentioning that the electrical conductivity of the permeate that was obtained for the different tests indicates that the fouling deposited on the membrane surface induced the leaking of the feed solution through the membrane to some extent. This leaking is clearly correlated to the pre-treatment. Permeate that was obtained without the pre-treatment of feed solution exhibited 8.6 μS cm^−1^, while the use of pre-treatment resulted in a permeation of 5.9 μS cm^−1^ for filtration and of 3.5 μS cm^−1^ for filtration and pH adjustment.

The material deposited on the membrane surface was preponderantly composed of Si, Fe, Al, Ca, Mg, and O. When filtration was employed as pre-treatment, the amounts of Si, Fe, and O in the fouling composition reduced, along with Ca and Mg. However, the fouling composition was absent of both Ca and Mg when filtration was associated with pH control. These results confirm that the precipitation of Ca- and Mg-based materials is suppressed when filtration is associated with pH adjustment. Microanalysis of the filtered materials reveals that the retained materials is mainly composed of Si, Fe, and Al, which corroborates with the microanalyses of the fouling deposited on membranes. An extensive discussion about the composition of fouling is found in our previous paper [7].

**Figure 6:**
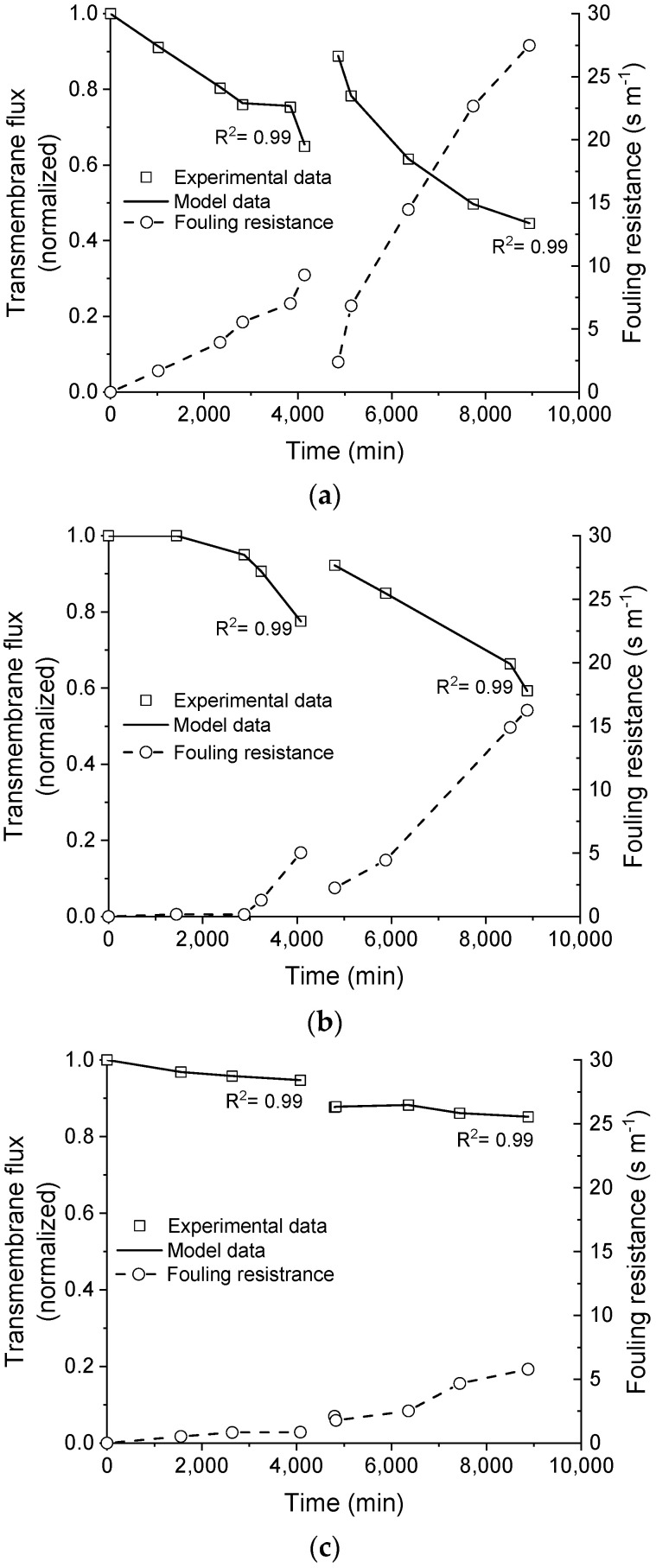
Transmembrane flux, predicted by the model fitted to the respective experimental data by adjusting the fouling resistance over time. Saline effluent as received (**a**), filtered (**b**), and filtered with pH adjusted (**c**).

The capability prediction of the developed model was improved by estimating some parameters to fit the experimental data. The adjustment of the transmembrane flux predicted by the model to the respective experimental data is plotted in Figure 6. As shown in the plots, the model is capable of predicting the transmembrane fluxes (line with squares in Figure 6), using the fouling resistance as a manipulated variable (dash with circle in Figure 6), as denoted by the coefficient (R^2^) obtained for the model fitting.

**Figure 7:**
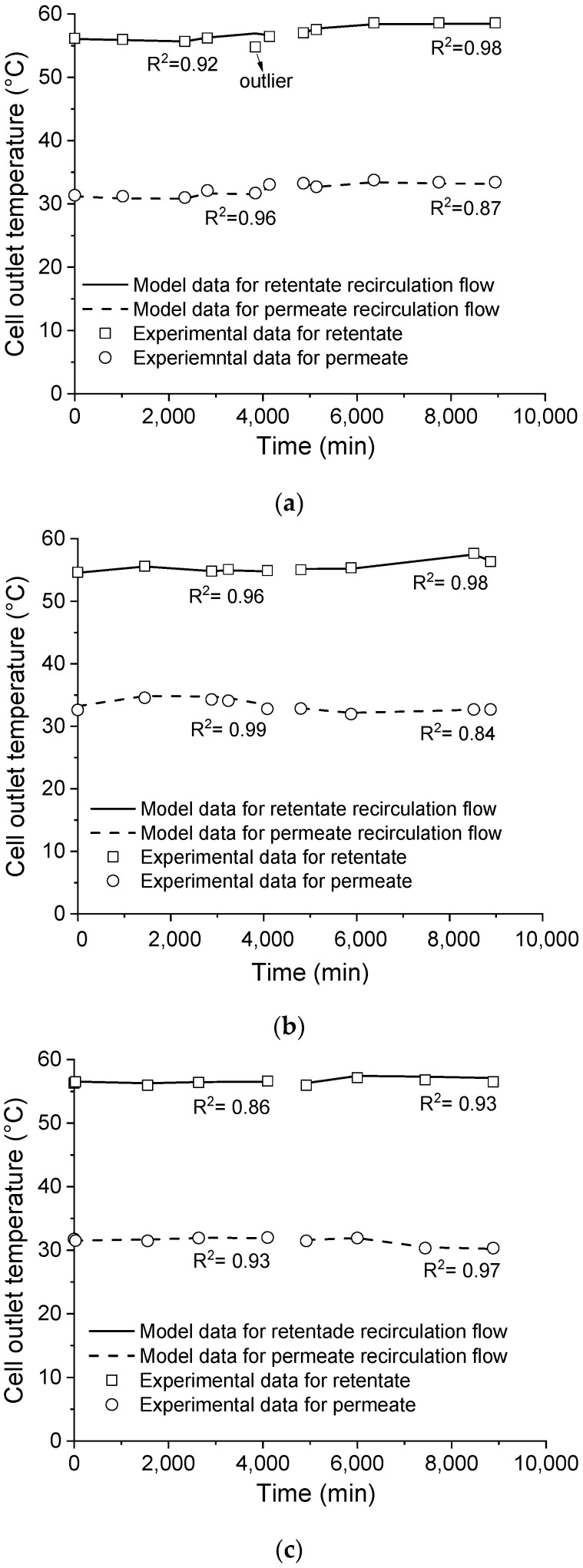
Comparison of the cell outlet temperatures predicted by the model with the respective experimental data. Saline effluent as received (**a**), filtered (**b**), and filtered with pH adjusted (**c**).

Indeed, the prediction capability of the model was also assessed by comparing the experimental and predicted outlet temperatures of both the retentate and permeate recirculating flows, which is shown in Figure 7. As seen, the coefficient of determination (R^2^) indicates a good fit between the model and experimental data and validates the model prediction capability. Besides, the model validation through both the transmembrane flux and the cell outlet temperatures ensures that the model assumptions for mass and thermal balances were taken properly and, consequently, the temperature and concentration on the membrane surface, which cannot be experimentally measured, were calculated accurately.

**Figure 8:**
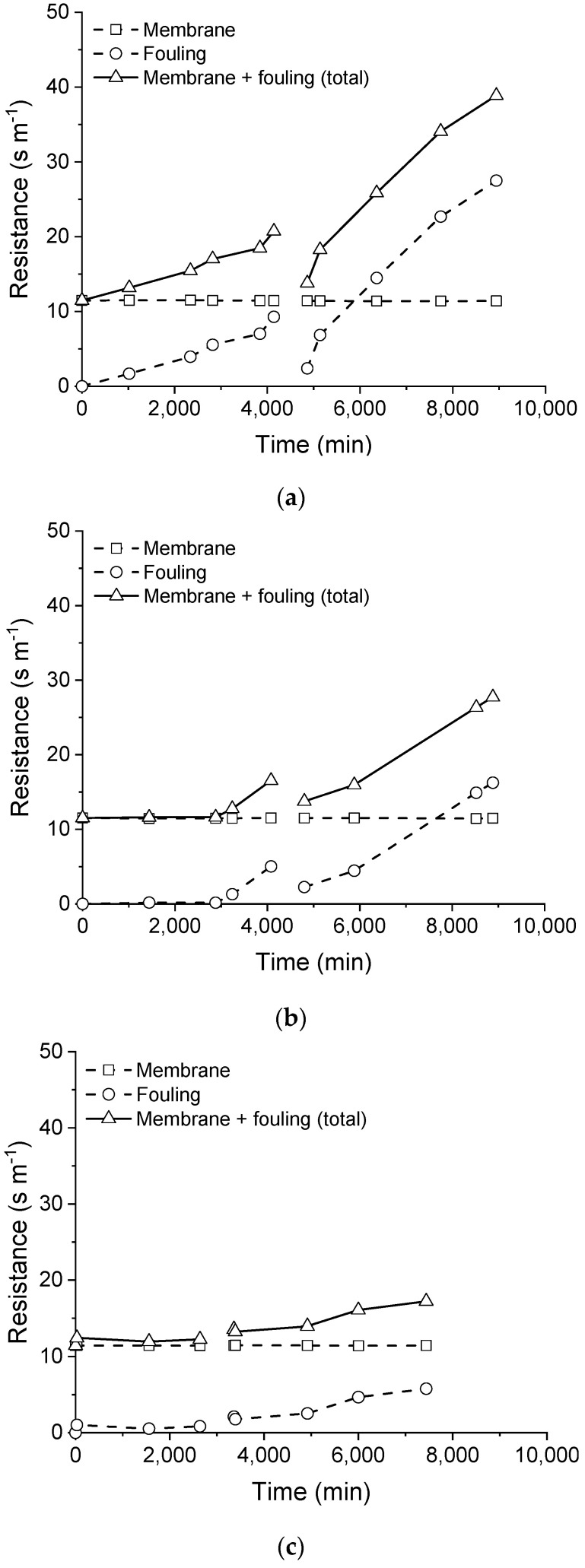
Resistances imposed by membrane and fouling. Saline effluent as received (**a**), filtered (**b**), and filtered with pH adjusted (**c**).

The resistances imposed by the membrane and by the fouling layer to the mass transfer are depicted in Figure 8. Regardless of the pre-treatment employed, the membrane resistance was about 11 s m^−1^, which is steady over the membrane distillation processing time. This result also indicates the consistency of the model prediction.

On the other hand, the resistance that was imposed by the fouling layer deposited on the membrane surface over time was influenced by the pre-treatment applied to the saline effluent, as indicated by the different profiles of the fouling resistance that were obtained for the tests (see Figure 8). A higher fouling resistance profile was obtained when the saline effluent was processed without any pre-treatment, reaching a maximum value of 30 s m^−1^ after the membrane cleaning. When filtration was employed, the fouling resistance profile was reduced in comparison with the processing of the saline as-received effluent, presenting a maximum value of 16 s m^−1^ after membrane cleaning. The processing of filtered and pH-adjusted saline effluent resulted in the lowest fouling resistance profile, with a maximum value of approximately 6 s m^−1^ after the membrane cleaning. From these results, the fouling resistance which was imposed to the water transported through the membrane when the pre-treatment of filtration was associated with an employed pH adjustment is 5-times lesser than the effluent without pre-treatment.

It is worth mentioning that, for the three experiments, the initial value of the fouling resistance was practically the same after the membrane cleaning (~2–3 s m^−1^), but higher than that calculated with the pristine membrane (zero). It corroborates with the conclusion that the fouling resistance created during processing was not eliminated by the cleaning procedure, and some materials remained on the membrane surface.

### 4.2. Dynamics of Fouling Deposition

The proposed dynamic model of fouling formation is expected to explain the fouling resistance profiles that were obtained from the experimentation and shown in Figure 8. The proposed fouling mechanism is based on the balance of the rates of particulate deposition, precipitated deposition, and entrainment, as described in Equations (1)–(7). The adjustment of the model-predicted fouling resistance to the respective regression data allows the obtention of the effective constants of the deposition rate of suspended particles (*K_part_* in Equation (1)), precipitates (*K_ppt_* in Equation (2)), and entrained material (*K_cis_* in Equation (3)). The regressed constants and the model fitting to the experimental data are depicted in Table 3.

The order of the deposition rate of precipitates (*n* in Equation (2)) was estimated as *n* = 2 by trial and error. As evidenced by the coefficient of determination (R^2^), the proposed model adequately fitted all the experimental data regardless of both the pre-treatment employed and the membrane’s surface condition, that is, pristine or cleaned. The distinct values of the effective constants found for each test indicate that the nature of the material that was deposited on the membrane and the interfacial properties of both membrane and foulants were affected by the pre-treatment of the feeding solution (Table 3). 

**Figure 9:**
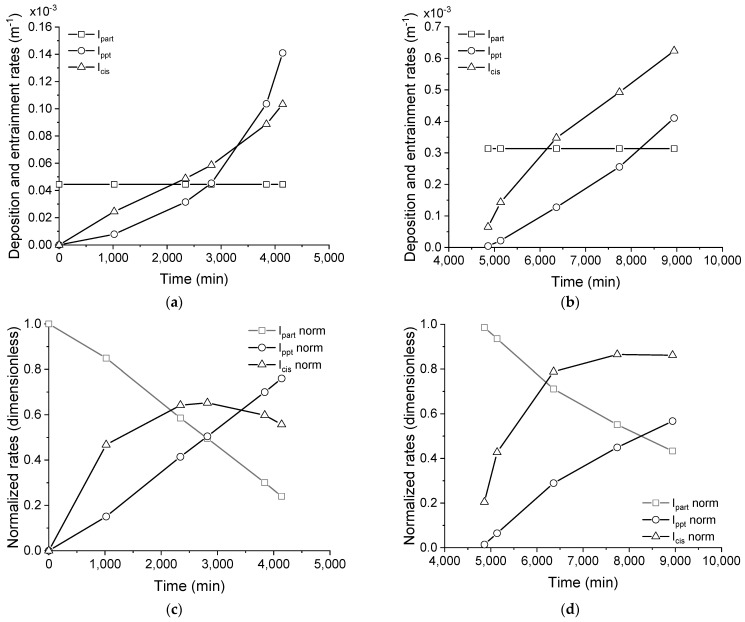
Rates of particulate and precipitate deposition and entrainment for the membrane distillation test processing the saline effluent as-received using pristine (**a**) and cleaned (**b**) membranes. Normalized rates (**c**,**d**).

The deposition rate of particulates and precipitates, in addition to the entrainment rate, are shown in Figure 9a,b for the membrane distillation test running the saline effluent as received, that is, without any pre-treatment. Additionally, the respective normalized rates are shown in Figure 9c,d for the sake of a better understanding of the fouling mechanism occurring with this effluent.

As seen in Figure 9a,b, the deposition of particulates and precipitates occurs concomitantly when processing the as-received effluent, using both the pristine and cleaned membranes. Indeed, the deposition rate of particulates was constant over time, while the deposition rate of precipitates increased exponentially by 4000 min. A higher deposition rate of particulates and precipitates, as well as entrainment rate, were found after the membrane cleaning procedure, as observed when comparing Figure 9a,b. The higher deposition rate of particulates could be explained by a change in the interfacial properties of the membrane that occurred after cleaning, which could favor the adherence of particles to the surface in the next processing round. This conclusion is corroborated by the higher *K_part_* that was obtained in the model regression for the process using a cleaned membrane (comparing *K_part_* in Table 3 for processing the as-received saline effluent). The higher deposition rate of precipitates obtained when processing with the cleaned, rather than pristine, membrane is attributed to the higher concentration of nucleation sites, which are formed by surface defects or even by deposited materials on the membrane, rather than the proportionality constant for precipitate fouling, which has a lower value instead (comparing *K_ppt_* in Table 3 for processing the as-received saline effluent). As indicated in Figure 9a,b, the deposition of particulates was higher than for precipitates until a crossover time at 3000 s, when the precipitate deposition started to be predominant. In this sense, an anisotropic fouling layer is formed over time which presents with, consequently, distinct interfacial properties.

**Figure 10:**
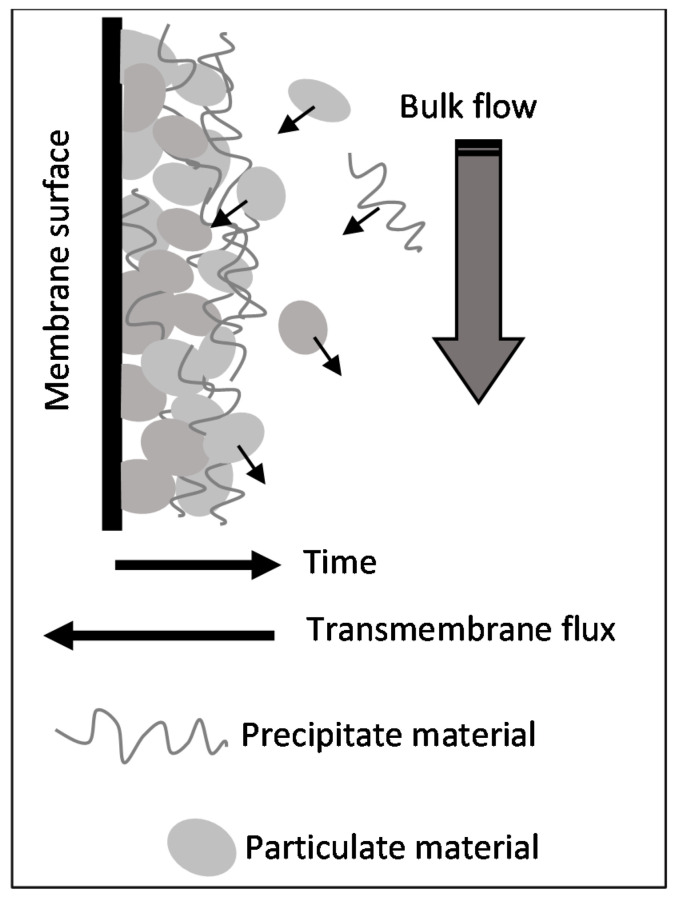
Mechanism of fouling formation occurring in the processing of concentrated saline effluent without pre-treatment by membrane distillation.

The normalized rates are shown in Figure 9c,d for the processing tests using the pristine and cleaned membranes, respectively. It is very important to note that the maximum entrainment rate occurred at approximately the crossover point and, from this moment on, started to decay, which is well-defined in Figure 9c. This behavior suggests that the deposits preponderantly formed by particulates tend to be weakly cohesive and, therefore, are more prone to entrain into the retentate bulk flow than the precipitate deposits. In other words, the precipitated material could serve as a coupling agent between adjacent particles, increasing the cohesion of the layer and, then, hampering the entrainment to the bulk flow. A scheme representing the proposed mechanism for fouling formation during the processing of the concentrate saline effluent without pre-treatment is shown in Figure 10.

**Figure 11:**
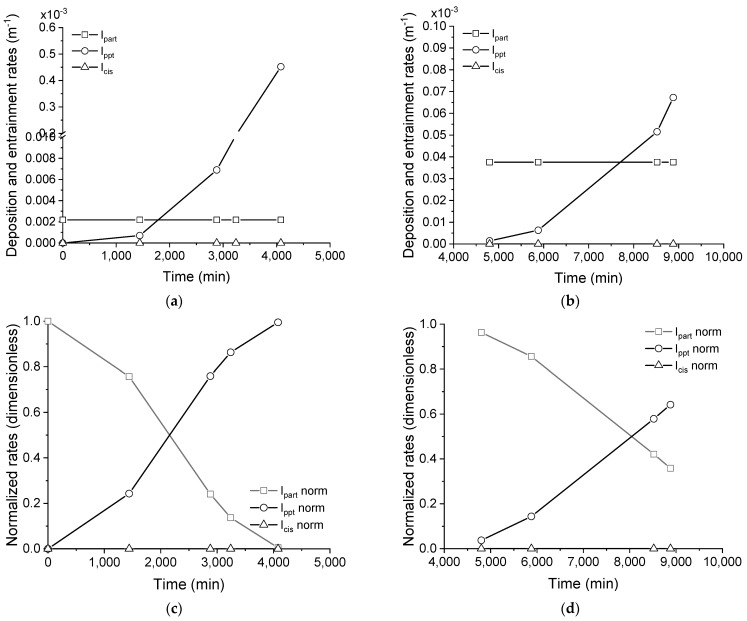
Rates of particulate and precipitate deposition and entrainment for the membrane distillation test, processing the filtered saline effluent using pristine (**a**) and cleaned (**b**) membrane. Normalized rates (**c**,**d**).

The behavior of fouling formation in the membrane distillation when the concentrated saline effluent was filtered differs from that without the pre-treatment, regardless of the nature of the membrane surface, that is, pristine or cleaned, as evidenced by the lower rates of particulate and precipitate depositions, as well as by the negligible rate of entrainment (compare Figure 9a,b and Figure 11a,b). The distinct behavior that was found when filtration was employed as a pre-treatment could be explained, considering the composition of the concentrated saline effluent. The particles that remained in the solution after filtration probably have interfacial properties that hampered their adherence to the membrane, as suggested by the lower proportionality constant (*K_part_*) in comparison with the effluent as-received for both the pristine and cleaned membranes (compare *K_part_* in Table 3). Additionally, the concentration of particles in solution after filtration is lower, which, in turn, intrinsically reduces the probability of deposition.

The deposition rate of the precipitates was featured by a latency period when a pristine membrane was employed in the processing (see Figure 11a), but not when a cleaned membrane was used (see Figure 11b). This latency time could be explained by the reduced concentration of potential sites for nucleation on the membrane. These nucleation sites could be formed by surface defects or even by deposited materials. The lower deposition rate of particulates could explain the existence of the latency period for the processing using the pristine membrane. On the other hand, the cleaning procedure was not able to remove the fouling that had formed in the previous membrane distillation processing and, therefore, some deposits remained on the membrane surface, which can play the role of nucleation sites, explaining the absence of a latency period when processing the filtered effluent using the cleaned membrane. At the same time, these residues probably favored the adherence of particulate fouling, as indicated by the higher *K_part_* that was obtained for the cleaned than pristine membrane (see *K_part_* in Table 3 for filtered effluent).

**Figure 12:**
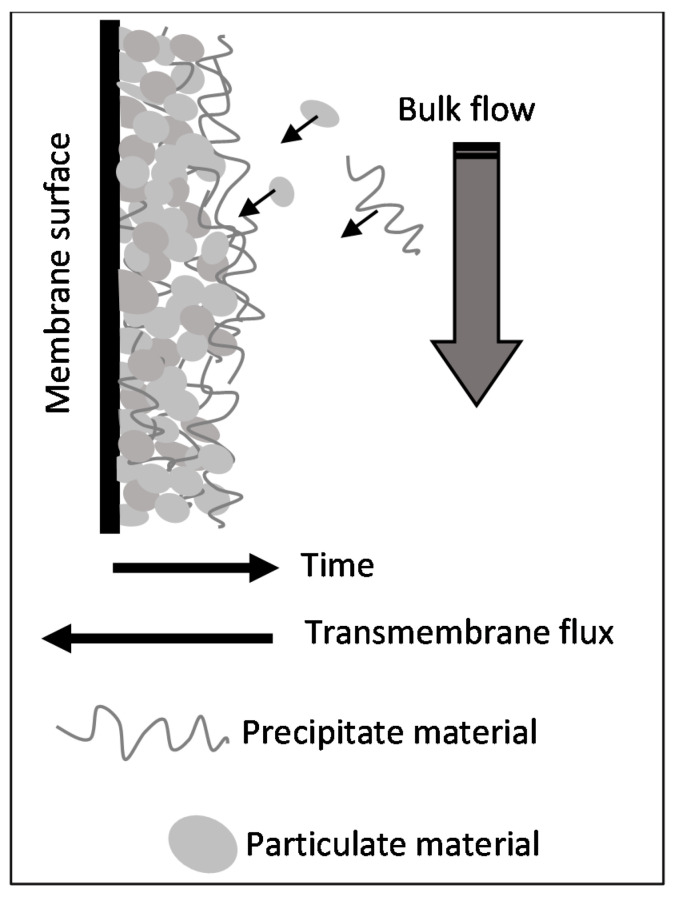
Mechanism of fouling formation occurring in the processing of filtered effluent by membrane distillation.

The negligible rate of entrainment suggests that the deposit layer which formed from the filtered effluent is cohesive, regardless of the process occurring with the pristine or cleaned membrane. This feature could be attributed to the nature of particles (small) and their interaction with the precipitate material. A scheme representing the proposed mechanism for fouling formation during the processing of the filtered effluent by membrane distillation is shown in Figure 12.

**Figure 13:**
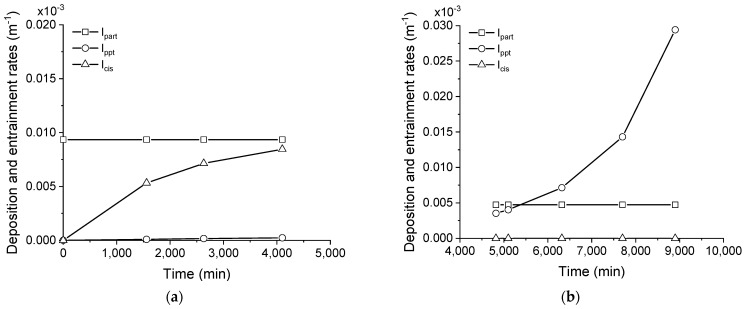

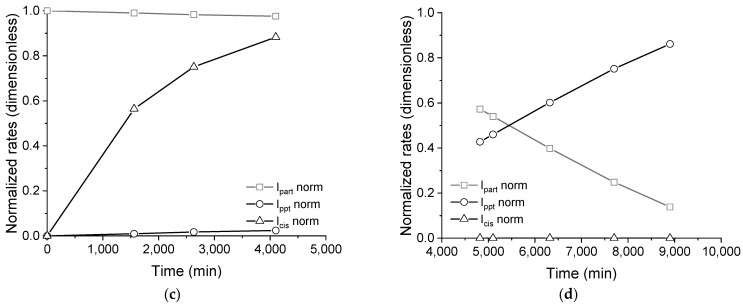
Rates of particulate and precipitate deposition and entrainment for membrane distillation test, processing the filtered + pH adjusted saline effluent using the pristine (**a**) and cleaned (**b**) membrane. Normalized rates (**c**,**d**).

The fouling formation was significantly reduced when the effluent was filtrated, and the solution pH was adjusted to about five during the membrane distillation test. The rates of deposition and entrainment obtained using the model reflect this experimental condition, as indicated in Figure 13a,b.

**Figure 14:**
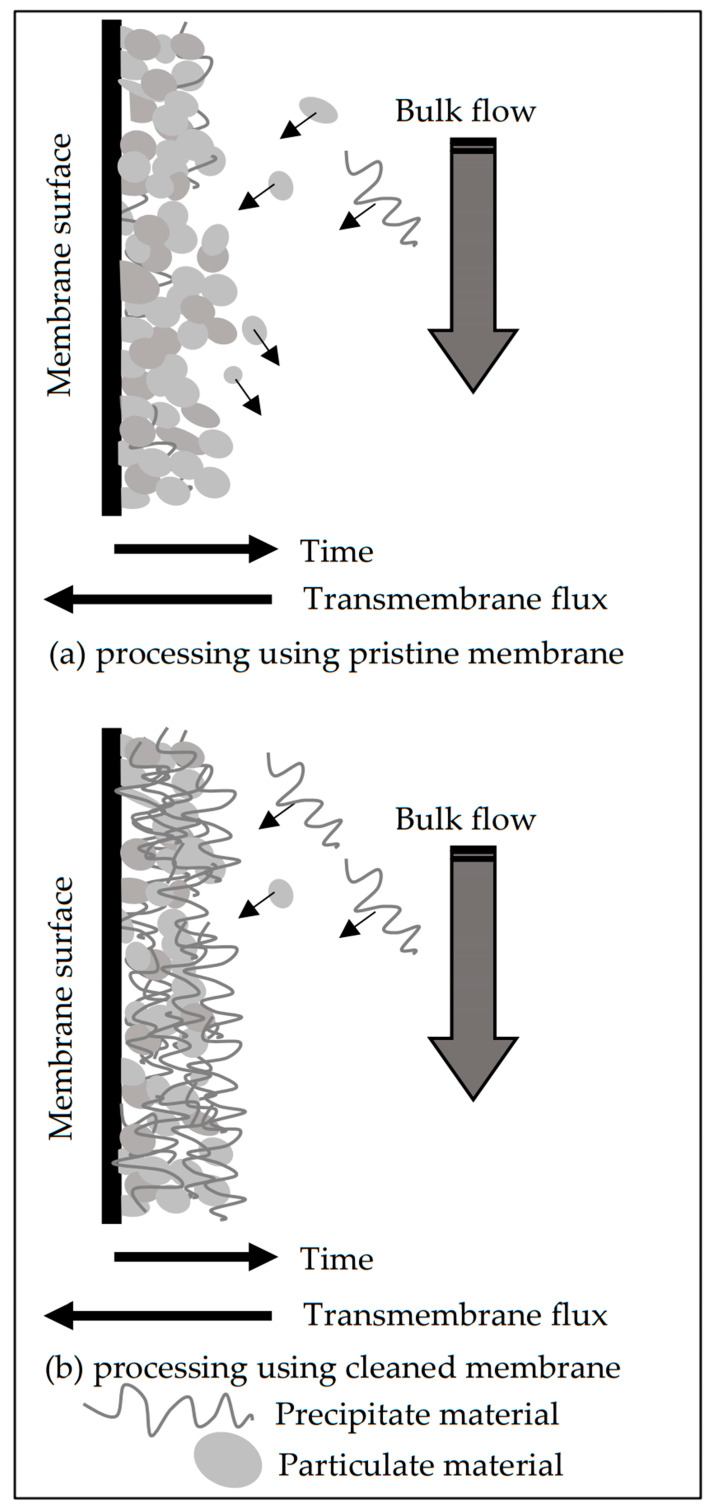
Mechanism of fouling formation occurring in the processing of filtered + pH adjusted effluent by membrane distillation with (**a**) pristine and (**b**) cleaned membrane.

The deposition rate of precipitates was suppressed when processing them using the pristine membrane, as evidenced in Figure 13a. This result was attributed to the maintenance of the solubility, due to the pH control over time, as well as to the low concentration of nucleation sites. However, when the processing occurred using the cleaned membrane, precipitate deposition appeared alongside particulate deposition (Figure 13b). This feature could be explained by the presence of residual deposits after the membrane cleaning, which served as nucleation sites for the precipitation. Also, it is not improbable that fluctuations of the pH over time can induce some precipitation. When the fouling layer was preponderantly formed by precipitates during the processing with the cleaned membrane (see Figure 13d), a negligible entrainment rate was obtained. Contrarily, when the fouling was formed mostly by particulates, which occurred when processing with the pristine membrane, the entrainment rate increased monotonically, as seen in Figure 13c, indicating the particulate layer that formed on the membrane’s surface was poorly cohesive. The distinct mechanisms of fouling formation, occurring when processing the filtered and pH adjusted effluent using the pristine membrane and cleaned membrane, are illustrated in Figure 14a,b, respectively.

## 5. Implications for the Membrane Distillation Technology

The results that were obtained in this study are limited to the kind of effluent employed. For effluents that can probably form particulate and precipitate fouling, the use of pre-treatments helps to increase the transmembrane flux. Through the mathematical modeling, it was possible to determine the predominant type of fouling, which, in turn, relies on the pre-treatment employed. The experiment carried out with the effluent, without pre-treatment, had fouling formed preferably by particulate material, while the experiment carried out with the filtered effluent had fouling formed preferably by precipitated material. With the pH adjustment of the filtered effluent, the formation of precipitates was avoided, and fouling was formed preferentially by particulate material. Based on these results, the design of a physico-chemical pre-treatment is imperative to maintain the operation of membrane distillation cells.

As suggested by the results, the particulate fouling exhibited a poor cohesion, and it is prone to entrainment. In this sense, a flow with high velocity profile over the membrane can help to control the fouling formation.

The rate of precipitate fouling was dependent on the availability of surface sites to anchor the precipitate formed in the solution. Indeed, even the remaining material on te membrane’s surface, due to a deficient cleaning procedure, lead to precipitate fouling. This result highlights the importance of a proper cleaning procedure to restore the performance capabilities of the membrane.

The results also indicated that the deposition rate increases after a membrane cleaning step. It is imperative to study the effects of repeated operation/cleaning/operation cycles on fouling mechanisms in further researches.

## 6. Conclusions

The experimental data that were obtained from the tests of membrane distillation, coupled with mathematical modeling, showed that the pre-treatments of filtrate or its association with the pH control over the processing time affects the mechanism and the dynamics of fouling that were obtained from concentrated saline effluent.

Based on the results described and discussed in the present research work, the fouling formation, occurring when processing concentrated saline effluent, could be mitigated by employing filtration and by adjusting the pH of the solution during the processing. The mitigation of precipitate fouling is preponderant to avoid the formation of a high-cohesive layer on the membrane. The proposed membrane cleaning procedure did not recover the membrane’s surface, and residues of fouling accelerated the formation of fouling in further tests.

It is important to recall that the knowledge about fouling mechanisms is essential to improve the design of membrane distillation units. Additionally, it seems that it is imperative to carry out further experimental tests to determine the fouling mechanism for a specific effluent, that is, there is no generic strategy to mitigate it.

## Figures and Tables

**Table 1:** Membrane technical datasheet [30].

Parameter	
Supplier	Clarcor
Model	Aspire^®^ QL217
Type	Hydrophobic
Functional layer	PTFE
Support layer	PP fibers
Thickness (μm)	150–250
Nominal pore size (μm)	0.2
Porosity (%)	80
Water entry pressure (barg)	≥1
Contact angle (°)	120
Filtration area (m^2^)	0.0132

**Table 2:** Average operation conditions of membrane distillations tests.

Parameter	Test 1	Test 2	Test 3
Inlet temperature of retentate flow (°C)	57.9	56.8	58.2
Outlet temperature of retentate flow (°C)	55.9	55.0	56.2
Inlet temperature of permeate flow (°C)	29.5	31.4	29.5
Outlet temperature permeate flow (°C)	31.7	33.7	31.6
Pressure of retentate flow (barg)	0.20	0.19	0.20
Pressure of permeate flow (barg)	0.05	0.06	0.05
Retentate flowrate (g s^−1^)	28		
Permeate flowrate (g s^−1^)	10		
Time (min)	9400		

Test 1: concentrated saline effluent as received; Test 2: filtered saline effluent; and Test 3: filtered + pH adjusted saline effluent.

**Table 3:** Fitting of the dynamic fouling model to the experimental data.

Effluent	Effective Kinetic Constant	Pristine Membrane	Cleaned Membrane	Model Adjust
Saline effluent as received	*K_part_*	26.33	185.55	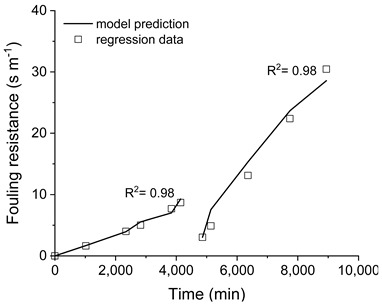
	*K_ppt_*	1.81 × 10^−6^	4.87 × 10^−7^	
	*K_cis_*	3.37 × 10^−2^	6.38 × 10^−2^	
Filtered saline effluent	*K_part_*	1.29	22.21	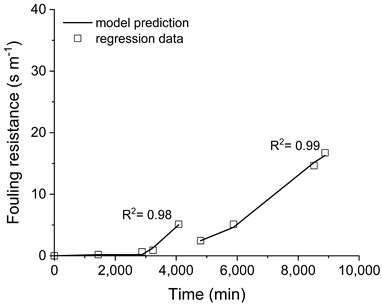
	*K_ppt_*	1.72 × 10^−5^	2.40 × 10^−7^	
	*K_cis_*	2.24 × 10^−8^	4.85 × 10^−9^	
Filtered + pH adjusted saline effluent	*K_part_*	5.53	2.80	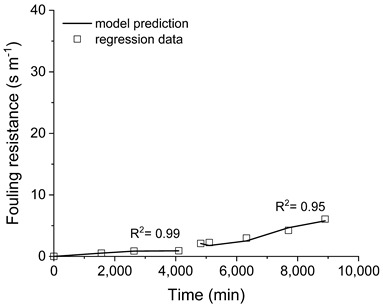
	*K_ppt_*	2.58 × 10^−7^	8.03 × 10^−7^	
	*K_cis_*	2.58 × 10^−2^	1.77 × 10^−9^	

## Data Availability

The data presented in this study are available on request from the corresponding author.

## Supplementary Materials

The following are available online at https://www.mdpi.com/article/10.3390/membranes11120958/s1, Table S1: Model parameters. References [30,38,39,40,41,42,43,44,45,46,47] are cited in Supplementary Materials.
