# Supplementary material for: Membrane Distillation: Pre-Treatment Effects on Fouling Dynamics
Source: Membranes (Basel). 2021 Dec 3;11(12):958. doi: 10.3390/membranes11120958 (PMC8706986; doi:10.3390/membranes11120958)
Supplement: Supplementary file 1 [file membranes-11-00958-s001.zip › membranes-1488527-supplementary.pdf]

membranes

Supplementary materials

Membrane Distillation: Pre-Treatment Effects on Fouling Dynamics

Paula G. Santos [1], Cíntia M. Scherer [2], Adriano G. Fisch [3,*] and Marco Antônio S. Rodrigues [4]

[1] Graduation Program in Environmental Quality, Universidade Feevale, 2755 ERS-239, Novo Hamburgo 93525-075, Brazil; paula.goncalves@feevale.br
[2] Chemical Engineering Department, Universidade Feevale, Novo Hamburgo 93525-075, Brazil; cintiascherer@feevale.br
[3] Chemical and Materials Engineering Department, University of Alberta, Edmonton, AB T6G 2R3, Canada
[4] Graduation Program in Technology of Materials and Industrial Processes, Universidade Feevale, Novo Hamburgo 93525-075, Brazil; marcor@feevale.br
* Correspondence: fisch@ualberta.ca

Abstract: In the research reported in this paper, membrane distillation was employed to recover water from a concentrated saline petrochemical effluent. According to the results, the use of membrane distillation is technically feasible when pre-treatments are employed to mitigate the fouling. Mathematical model was used to evaluate the fouling mechanism showing that deposition of particulate and precipitated material occurs in all tests, however, the fouling dynamic depends on the pre-treatment employed (filtration, or filtration associated with pH adjustment). The deposit layer formed by particles is not cohesive allowing its entrainment to the bulk flow. The precipitate fouling showed a minimal tendency to entrainment. Also, precipitate fouling served as a coupling agent among adjacent particles increasing the fouling layer cohesion.

Keywords: membrane distillation; fouling; petrochemical effluent; pre-treatment; water recovery

Citation: Santos, P.G.; Scherer, C.M.; Fisch, A.G.; Rodrigues, M.A.S. Membrane Distillation: Pre-Treatment Effects on Fouling Dynamic. *Membranes* **2021**, *11*, 958. https://doi.org/10.3390/membranes11120958

Academic Editors: Szilárd S. Bucs, Nadia Farhat and Luca Fortunato

Received: 16 November 2021
Accepted: 30 November 2021
Published: 3 December 2021

Publisher's Note: MDPI stays neutral with regard to jurisdictional claims in published maps and institutional affiliations.

Copyright: © 2021 by the authors. Licensee MDPI, Basel, Switzerland. This article is an open access article distributed under the terms and conditions of the Creative Commons Attribution (CC BY) license (https://creativecommons.org/licenses/by/4.0/).

1. Mathematical modeling

1.1. Transmembrane flux

Transmembrane flux is evaluated by Equation (1), in which the vapor flow throughout the membrane pores is assumed to occur throughout a stagnant medium, that is, Stefan's flow [1,2].

$$J_A = \frac{1}{R_t} c \, ln\left(\frac{1 - y_{A,p}}{1 - y_{A,f}}\right) \quad (1)$$

where: J_A is the transmembrane flux (kg m^{-2} s^{-1}), c is the total concentration of the system (kg m^{-3}), $y_{A,f}$ is the molar fraction of water vapor in the feed side (retentate), and $y_{A,p}$ is the molar fraction of water vapor in the permeate side (distillate).

O term R_t (s m^{-1}) in Equation (1) is the resistance imposed by the medium on the solute transport, which is the summation of that imposed by the membrane (R_m) and by the eventual fouling (R_f), as given in Equation (2).

$$\frac{1}{R_t} = \frac{1}{R_m + R_f} = \frac{1}{\frac{z}{D_{Am}} + R_f} \quad (2)$$

where: R_t is the total resistance imposed on the transport (s m^{-1}), R_m is the resistance imposed by the membrane (s m^{-1}), R_f is the resistance imposed by fouling (s m^{-1}), z is the membrane thickness (m), and $D_{A,m}$ is the diffusion coefficient (m^2 s^{-1}) which is assessed as given in Equations (3)–(8).

The mass flux throughout a microporous membrane is an association of advective and diffusive mechanisms. The diffusion is dependent on the material morphology, mainly on its porosity and tortuosity [1], as described in Equation (3).

$$D_{A,m} = \frac{\phi}{\tau} D_A \tag{3}$$

Where: $D_{A,m}$ is the effective diffusion coefficient, ϕ is the material porosity, and τ is the pore tortuosity. D_A is the ordinary or Knudsen, or even an association of both, diffusion coefficient, which depends on the average pore size of the material by definition.

The Knudsen diffusion features a higher collision probability of diffusants with the pore walls than intramolecular collisions as the molecule mean free path (λ_I) is longer than the average pore size (d_p) [2]. Thereby, the Knudsen number, which is defined in Equation (4), is used to evaluate the implication of the average pore size on the diffusion mechanism [1].

$$N_{Kn} = \frac{\lambda_i}{d_p} \tag{4}$$

$$\lambda_i = 3.08 \times 10^{-7} \frac{T}{\sigma_i P} \tag{5}$$

where: N_{Kn} is the Knudsen number (dimensionless), λ_i is the mean free path of molecule *i* (cm), *T* is the temperature (K), σ_i is the collision diameter (Å), d_p is the average pore size (cm), and *P* is the absolute pressure (atm).

If $N_{kn} < 0.1$, the ordinary diffusion dominates the transport mechanism, which considers the diffusion of water vapor through the air confined inside the pores [2], as given in Equation (6).

$$D_A = D_{AB} = 1{,}87 \times 10^{-10} \frac{T^{2.072}}{P} \tag{6}$$

where: D_{AB} is the ordinary diffusion coefficient ($m^2\ s^{-1}$), *T* is the temperature (K), and *P* is the absolute pressure (atm).

If $N_{Kn} > 10$, Knudsen diffusion dominates the transport mechanism. The diffusion coefficient is estimated as given in Equation (7),

$$D_A = D_{A,kn} = 0.485 d_p \left(\frac{T}{M_A}\right)^{0{,}5} \tag{7}$$

where: $D_{A,kn}$is the Knudsen diffusion coefficient ($m^2\ s^{-1}$), *T* is the temperature (K), d_p is the average pore size (cm), and M_A is the molar mass of the diffusant A ($g\ mol^{-1}$).

Now, if $0.1 < N_{kn} < 10$, an associative mechanism dominates the transport. In such a case, the effective diffusion coefficient ($D_{A,m}$) is an average between the ordinary (D_{AB}) and Knudsen ($D_{A,kn}$) diffusion coefficients, as given in Equation (8).

$$\frac{1}{D_A} = \frac{1}{D_{AB}} + \frac{1}{D_{A,kn}} \tag{8}$$

where: D_A is the diffusion coefficient, D_{AB} is the ordinary diffusion coefficient, and $D_{A,kn}$ is the Knudsen diffusion coefficient.

The molar fraction of water vapor in opposite sides of the membrane, $y_{A,f}$ and $y_{A,p}$, are evaluated considering a non-ideal solution model [3], as given in Equations (9) and (10), respectively.

$$y_{A,f} = \frac{p_{v,f}}{P} a_{w,f} \tag{9}$$

$$y_{A,p} = \frac{p_{v,p}}{P} a_{w,p} \tag{10}$$

where: $y_{A,f}$ and $y_{A,p}$ are the molar fractions of water vapor, $p_{v,f}$ and $p_{v,p}$ are the vapor pressures of water (Pa), $a_{w,f}$ and $a_{w,p}$ are the activities of water, and P is the total pressure (Pa). Sub-indexes f and p mean data from feed (retentate) and permeate solution, respectively.

The vapor pressure of water in the retentate and permeate cell side are predicted by Antoine's equation as given in Equations (11) and (12), respectively,

$$p_{v,f} = e^{A - \frac{B}{C + T_m}} \tag{11}$$

$$p_{v,p} = e^{A - \frac{B}{C + t_m}} \tag{12}$$

where: $p_{v,f}$ and $p_{v,p}$ are the vapor pressure of water (Pa) for retentate and permeate solutions, respectively, T_m and t_m are the temperature of membrane surface (K) for retentate and permeate sides, respectively, A, B, and C are Antoine's constants for water (A = 231963, B = 3816.44, and C = −46.13) [3,4].

1.2. Temperature polarization

The temperature polarization coefficient is assessed by Equation (13) [5,6].

$$PTC = \frac{T_m - t_m}{\overline{T} - \overline{t}} \tag{13}$$

where: PTC is the polarization temperature coefficient, $\overline{T}$is the logarithmic mean of the bulk temperature of retentate solution (K), $\overline{t}$ is the logarithmic mean of the bulk temperature of permeate solution (K), T_m is the temperature of membrane surface of retentate side (K), and t_m is the temperature of membrane surface of the permeate side (K).

The temperature of the membrane surface of the retentate side (T_m) is calculated by Equation (14).

$$T_m = \overline{T} - \frac{Q_{evap} + Q_{cm}}{h_f A} \tag{14}$$

where: T_m is the temperature of the membrane surface of the retentate side (K), $\overline{T}$ is the logarithmic mean of the bulk temperature of retentate solution (K), Q_{evap} is the heat required for water evaporation (W), Q_{cm} is the heat that is transferred from the retentate side for the permeate by conduction through the membrane (W), h_f is the convective heat transfer coefficient of retentate side (W m^{-2} K^{-1}), and A is the transversal area of the membrane for heat flux (m^2).

The temperature of the membrane surface of the permeate side is calculated by Equation (15).

$$t_m = \overline{t} + \frac{Q_{evap} + Q_{cm}}{h_p A} \tag{15}$$

onde: t_m is the temperature of membrane surface of the permeate side (K), $\overline{t}$ is the logarithmic mean of the bulk temperature of permeate solution (K), Q_{evap} is the heat required for water evaporation (W), Q_{cm} is the heat that is transferred from the retentate side for the permeate by conduction through the membrane (W), h_p is the convective heat transfer coefficient of permeate side (W m^{-2} K^{-1}), and A is the transversal area of the membrane for heat flux (m^2).

The convective heat transfer coefficient of retentate and permeate sides are calculated by Equations (16) and (17), respectively.

$$h_f = \frac{k_f}{D_h} N_{Nu,f} \tag{16}$$

$$h_p = \frac{k_p}{D_h} N_{Nu,p} \tag{17}$$

where: h_f and h_p are the convective heat transfer coefficient (W m^{-2} K^{-1}) of retentate and permeate side, respectively, k_f and k_p are the thermal conductivity (W m^{-1} K^{-1}) of the retentate and permeate solutions, respectively, D_h is the hydraulic diameter of the flow channel in the cell (m), and $N_{Nu,f}$ and $N_{Nu,p}$ are the Nusselt number of the retentate and permeate streams, respectively.

The Nusselt number is calculated by Equations (18) and (19) considering an internal flow in non-circular channels with a correction regarding the cell geometry (f_M factor) for reduced Reynolds numbers [7].

$$N_{Nu,f} = \begin{cases} f_M N_{Re,f}{}^{\frac{1}{3}} 1.86 \left(\frac{N_{Re,f} N_{Pr,f} D_h}{L}\right)^{\frac{1}{3}} \rightarrow N_{\mathrm{Re}}<10{,}000 \\ 0.023 N_{Re,f}{}^{0,8} N_{Pr,f}{}^{0,3} \rightarrow N_{\mathrm{Re}} \geq 10{,}000 \text{ e } 0.7 \leq N_{\mathrm{Pr}} \leq 16{,}700 \end{cases} \quad (18)$$

$$N_{Nu,p} = \begin{cases} f_M N_{Re,p}{}^{\frac{1}{3}} 1.86 \left(\frac{N_{Re,p} N_{Pr,p} D_h}{L}\right)^{\frac{1}{3}} \\ 0.023 N_{Re,p}{}^{0.8} N_{Pr,p}{}^{0.3} \end{cases} \quad (19)$$

where: $N_{Nu,f}$ and $N_{Nu,p}$ are the Nusselt number of the retentate and permeate streams, respectively, $N_{Re,f}$ and $N_{Re,p}$ are Reynolds number of retentate and permeate streams, respectively, and $N_{Pr,f}$ and $N_{Pr,p}$ are the Prandtl number of retentate and permeate streams, respectively.

The Reynolds number is assessed by Equations (20) and (21), respectively, for retentate and permeate streams.

$$N_{Re,f} = \frac{G_f D_h}{A_s \mu_f} \quad (20)$$

$$N_{Re,p} = \frac{G_p D_h}{A_s \mu_p} \quad (21)$$

where: G_f and G_p are the mass flow rate (kg s^{-1}) of retentate and permeate streams, respectively, μ_f and μ_p are the dynamic viscosity (kg m^{-1} s^{-1}) of retentate and permeate solutions, respectively, D_h is the hydraulic diameter (m), and A_s is the area (m^2).

Prandtl number is assessed by Equations (22) and (23), respectively, for retentate and permeate streams.

$$N_{Pr,f} = \frac{c_{p,f} \mu_f}{k_f} \quad (22)$$

$$N_{Pr,p} = \frac{c_{p,p} \mu_p}{k_p} \quad (23)$$

where: $c_{p,f}$ and $c_{p,p}$ are the specific heat (J kg^{-1} K^{-1}) of retentate and permeate streams, μ_f and μ_p are the dynamic viscosity (kg m^{-1} s^{-1}) of retentate and permeate solutions, respectively, and k_f and k_p are the thermal conductivity (W m^{-1} K^{-1}) of retentate and permeate solutions, respectively.

The rate of heat due to the water evaporation is dependent on the transmembrane flow, as given in Equation (24).

$$Q_{evap} = J_A A \Delta H_{evap} \quad (24)$$

where: Q_{evap} is the evaporation heat rate (W), J_A is the transmembrane flux (kg m^{-2} s^{-1}), A is the filtration area (m^2), and ΔH_{evap} is the heat of water evaporation (J kg^{-1}).

The rate of heat transferred by conduction through the membrane is calculated by the Equation (25)

$$Q_{cm} = \frac{k_m}{z} A (T_m - t_m) \quad (25)$$

where: Q_{cm} is the rate of heat conducted through the membrane (W), k_m is the conductivity of the membrane (W m^{-1} K^{-1}), A is the filtration area (m^2), z is the thickness of the membrane (m), T_m and t_m are, respectively, the temperature at the membrane surface of the retentate side and permeate (K).

The rate of heat lost to the environment due to poor insulation (W) is calculated in Equations (26) and (27) for retentate and permeate cell side, respectively.

$$Q_{l,f} = (U_f A)(\overline{T} - T_{amb}) \tag{26}$$

$$Q_{l,p} = (U_p A)(\overline{t} - T_{amb}) \tag{27}$$

where: ($U_f A$) and ($U_p A$) are, respectively, the global coefficient of heat exchange for the retentate and permeate side, $\overline{T}$and $\overline{t}$ are, respectively, the mean temperature of the retentate and permeate streams (K), and T_{amb} is the room temperature (K).

The mean temperature of the retentate and permeate streams is calculated as the logarithmic mean between the respective inlet and outlet temperatures of the cell, according to the Equations (28) and (29), respectively.

$$\overline{T} = \frac{(T_i - T_o)}{log(T_i - T_o)} \tag{28}$$

$$\overline{t} = \frac{(t_i - t_o)}{log(t_i - t_o)} \tag{29}$$

where: $\overline{T}$ and $\overline{t}$ are, respectively, the mean temperature of the retentate and permeate streams (K), T_i and t_i are, respectively, the inlet temperature of the retentate and permeate streams (K), and T_o and t_o are, respectively, the outlet temperature of the retentate and permeate streams (K).

The outlet temperature of the retentate and permeate streams are calculated from the energy balance in the respective cell sides (Equations (30) and (31), respectively).

$$T_o = T_i - \frac{Q_{evap} + Q_{cm} + Q_{l,f}}{G_f c_{p,f}} \tag{30}$$

$$t_o = t_i + \frac{Q_{evap} + Q_{cm} - Q_{l,p}}{G_p c_{p,p}} \tag{31}$$

where: T_i and t_i are, respectively, the inlet temperature of the retentate and permeate streams (K), T_o and t_o are, respectively, the outlet temperature of the retentate and permeate streams (K), G_f and G_p are, respectively, the mass flow rate of the retentate and permeate streams (kg s^{-1}), $c_{p,f}$ and $c_{p,p}$ are the specific heat of the retentate and permeate streams, respectively (J kg^{-1} K^{-1}), Q_{evap} is the rate of heat required for water evaporation (W), Q_{cm} is the rate of heat conducted through the membrane (W), and $Q_{l,f}$ and $Q_{l,p}$ are, respectively, the rate of heat lost to the environment due to poor insulation in the retentate and permeate cell side.

1.3. Concentration polarization

The concentration polarization coefficient, which is defined as the ratio between the concentration of solute in the retentate stream and that on the membrane surface, is calculated according to Equation (32) [4,6,8]. Diffusion and advection are the main mechanisms for transporting water through the boundary layer and were considered for obtaining Equation (32).

$$CPC = \frac{c_m}{c} = exp\left(\frac{J}{h_{massa}\rho_f}\right) \tag{32}$$

where: CPC is the concentration polarization coefficient, c_m is the concentration of solute on the membrane surface (kg m^{-3}), c is the concentration of solute in the retentate streams (kg m^{-3}), J is the transmembrane flux (kg m^{-2} s^{-1}), h_{massa} is the convective mass

transfer coefficient in the boundary layer of the retentate flow over membrane surface (m s^{-1}), and ρ_f is the density of the retentate solution (kg m^{-3}).

Considering the Chilton-Colburn analogy [2], the convective mass transfer coefficient in the boundary layer (h_{massa}) can be estimated from the respective convective heat transfer coefficient (h_f in Equation (16)), as expressed in Equation (33).

$$h_{massa} = \frac{h_f}{\rho_f c_{p,f} \left(\frac{N_{Sc,f}}{N_{Pr,f}}\right)^{\frac{2}{3}}} \tag{33}$$

where: h_{massa} is the convective mass transfer coefficient in the boundary layer of the retentate stream (m s^{-1}), h_f is the convective heat transfer coefficient (W m^{-2} K^{-1}), ρ_f is the density of the retentate solution (kg m^{-3}), $c_{p,f}$ is the specific heat of the retentate solution (J kg^{-1} K^{-1}), $N_{Sc,f}$ is the Schmidt number of the retentate stream, and $N_{Pr,f}$ is the Prandtl number of the retentate stream.

The Schmidt number is defined in Equation (34).

$$N_{Sc,f} = \frac{\mu_f}{\rho_f D} \tag{34}$$

where: N_{Sc} is the Schmidt number, μ_f is the viscosity of the retentate solution (kg m^{-1} s^{-1}), ρ_f is the specific mass of the water (kg m^{-3}) and D is the diffusivity coefficient of the solution of feeding (m^2 s^{-1}).

1.4. Module dimensions and other physical and physical-chemical properties

The hydraulic diameter of a rectangular duct (D_h) is calculated by Equation (35) [2].

$$D_h = \frac{2ab}{a+b} \tag{35}$$

where: a and b are the cell height and width, respectively (m).

The cross-sectional area that is available for the flow of the retentate and permeate streams (A_s) is calculated by Equation (36).

$$A_s = ab \tag{36}$$

where: a and b are the cell dimensions (m).

The membrane filtration area (A) is given by Equation (37).

$$A = Lb \tag{37}$$

where: b is the cell width (m) and L is the cell length (m).

The diffusion coefficient of the retentate stream (D) was assessed as that of the self-diffusion of water and estimated by the correlation of Equation (38) [9].

$$D_w = 1.1073 \times 10^{-9} + 3.91 \times 10^{-11}\overline{T_\delta} + 4.00 \times 10^{-13}\overline{T_\delta}^2 \tag{38}$$

where: D_w is the water self-diffusion coefficient (m^2 s^{-1}) and $\overline{T_\delta}$ is the arithmetic mean of the temperature in the boundary layer (K), that is, $\overline{T_\delta} = 0{,}5\left(T_m + \overline{T}\right)$.

The specific heat of the retentate ($C_{p,f}$) and permeate ($C_{p,p}$) solution can be approximated to that of the liquid water and, therefore, it can be estimated as a function of temperature, as given in Equation (39) [2].

$$c_{p,w} = 4.58772 \times 10^4 - 4.98678 \times 10^2\overline{T} + 2.23642\overline{T}^2 - 4.46118 \times 10^{-3}\overline{T}^3 + 3.34301 \times 10^{-6}\overline{T}^4 \tag{39}$$

where: $c_{p,w}$ is the specific heat (J kg^{-1} K^{-1}) and $\overline{T}$ is the logarithmic mean between the inlet and outlet temperatures of the stream (K).

The thermal conductivity of the retentate (k_f) and permeate (k_p) solutions can be approximated to that of liquid water and, therefore, it can be estimated as a function of temperature, as given in Equation (40) [2].

$$k_w = -7.59272 \times 10^{-1} + 7.49429 \times 10^{-3}\overline{T} - 9.76017 \times 10^{-6}\overline{T}^2 \quad (40)$$

where: k_w is the thermal conductivity of liquid water (W m^{-1} K^{-1}) and $\overline{T}$is the logarithmic mean between the inlet and outlet temperatures of the stream (K).

The viscosity of the retentate (μ_f) and permeate (μ_p) solutions can be approximated to that of liquid water and, therefore, it can be estimated as a function of temperature, as given in Equation (41) [2].

$$\mu_w = 2.70607 \times 10^{-2} - 1.53146 \times 10^{-4}\overline{T} + 2.19785 \times 10^{-7}\overline{T}^2 \quad (41)$$

where: μ_w is the dynamic viscosity of liquid water (kg m^{-1} s^{-1}) and $\overline{T}$is the logarithmic mean between the inlet and outlet temperatures of the stream (K).

The density of the retentate solution (ρ_f) was approximated as that of liquid water and, therefore, it can be estimated as a function of temperature, as given by Equation (42) [10].

$$\rho_w = 999.85308 + 6.32693 \times 10^{-2}\overline{T_\delta} - 8.523829 \times 10^{-3}\overline{T_\delta}^2 + 6.943248 \times 10^{-5}\overline{T_\delta}^3 - 3.821216 \times 10^{-7}\overline{T_\delta}^4 \quad (42)$$

where: ρ_w is the density of water (kg m^{-3}) and $\overline{T_\delta}$is the arithmetic mean of the temperature in the boundary layer (° C), $\overline{T_\delta} = 0{,}5\left(T_m + \overline{T}\right)$.

The heat of water evaporation is predicted by Equation (43) [2].

$$\Delta H_{evap} = 1.7535 \times 10^3 T_m + 2.0243 \times 10^6 \quad (43)$$

where: T_m is the temperature at the membrane surface on the retentate side (K) and ΔH_{evap} is the heat of water evaporation (J kg^{-1}).

2. Implementation of the model

The model has several parameters that must have their values fixed during the simulation. These parameters are listed in Table S1.

Table S1. Model parameters.

	Parameter	Value	Unit	Ref.
Membrane and cell data	Membrane porosity	0.8	-	[11]
	Membrane tortuosity	1.04	-	
	Membrane thickness	150	μm	
	Average pore size	0.2	μm	
	Cell thickness	1	cm	This work
	Cell length	14	cm	
	Cell height	9.5	cm	
Process data	Flowrate of retentate	1.69	kg min^{-1}	This work
	Flowrate of permeate	0.59	kg min^{-1}	
	Room temperature	298 (25)	K (°C)	
	Overall heat transfer coefficient for retentate side	2.5	W K^{-1}	This work (try-and-error adjust)
	Overall heat transfer coefficient for permeate side	3.2	W K^{-1}	
	Cell factor for Nusselt correction	0.5017	-	[7]
Physical-chemical data	Water molar mass	18	g mol^{-1}	[1,2]
	Collision diameter	2.649	Å	
	Concentration in the membrane pores	40.8969	mol m^{-3}	[3,4]
	Antoine constant, A	23.1963	-	
	Antoine constant, B	3,816.44	-	
	Antoine constant, C	−46.13	-	

Water activity	0.9	-	This work

References

1. Martynenko, O.G.; Pavlyukevich, N.V. Heat and mass transfer in porous media. *J. Eng. Phys. Thermophys.* **1998**, *71*, 1–13, https://doi.org/10.1007/bf02682488.
2. Çengel, Y.A.; Ghajar, A.J. *Heat and mass transfer fundamentals and applications*, 4th ed., McGraw-Hill: New York, NY, USA, 2011; pp .
3. Koretsky, M.D. *Engineering and chemical thermodynamics*, 2nd ed., John Wiley & Sons: New York, NY, USA, 2012; pp.
4. Curcio, E.; Drioli, E. Membrane Distillation and Related Operations—A Review. *Sep. Purif. Rev.* **2005**, *34*, 35–86, https://doi.org/10.1081/spm-200054951.
5. Nariyoshi, Y.N. Estudo dos fundamentos de cristalização assistida por destilação com membranas em aplicação de dessalinização de água, Ph.D. Thesis, University of São Paulo, São Paulo, Brazil, 2016.
6. Olatunji, S.O.; Camacho, L.M. Heat and Mass Transport in Modeling Membrane Distillation Configurations: A Review. *Front. Energy Res.* **2018**, *6*, https://doi.org/10.3389/fenrg.2018.00130.
7. Leitch, M.E.; Lowry, G.V.; Mauter, M.S. Characterizing convective heat transfer coefficients in membrane distillation cassettes. *J. Membr. Sci.* **2017**, *538*, 108–121, https://doi.org/10.1016/j.memsci.2017.05.028.
8. Martínez-Díez, L.; Vázquez-González, M.I. Temperature and concentration polarization in membrane distillation of aqueous salt solutions. *J. Membr. Sci.* **1999**, *156*, 265–273, doi:10.1016/s0376-7388(98)00349-4.
9. Easteal, A.J.; Price, W.E.; Woolf, L.A. Diaphragm cell for high-temperature diffusion measurements. Tracer Diffusion coefficients for water to 363 K. *J. Chem. Soc. Faraday Trans. 1: Phys. Chem. Condens. Phases* **1989**, *85*, 1091–1097, https://doi.org/10.1039/f19898501091.
10. Jones, F.; Harris, G.L. ITS-90 density of water formulation for volumetric standards calibration. *J. Res. Natl. Inst. Stand. Technol.* **1992**, *97*, 335–340, https://doi.org/10.6028/jres.097.013.
11. Dyrda, K.M.; Wilke, V.; Haas-Santo, K.; Dittmeyer, R. Experimental Investigation of the Gas/Liquid Phase Separation Using a Membrane-Based Micro Contactor. *ChemEngineering* **2018**, *2*, 55. https://doi.org/10.3390/chemengineering2040055.
